# An outbreak of cryptosporidiosis associated with drinking water in north-eastern Italy, August 2019: microbiological and environmental investigations

**DOI:** 10.2807/1560-7917.ES.2022.27.35.2200038

**Published:** 2022-09-01

**Authors:** Armando Franceschelli, Lucia Bonadonna, Simone M Cacciò, Anna Rosa Sannella, Christian Cintori, Raffaele Gargiulo, Anna Maria Coccia, Rosa Paradiso, Marcello Iaconelli, Rossella Briancesco, Alberto Tripodi

**Affiliations:** 1 Food Hygiene and Nutrition Service, Public Health Department, Local Health Unit of Modena, Modena, Italy; 2 Department of Environment and Health, Istituto Superiore di Sanità, Rome, Italy; 3 Department of Infectious Diseases, Istituto Superiore di Sanità, Rome, Italy; 4 Public Hygiene Service, Public Health Department, Local Health Unit of Modena, Modena, Italy; 5 Prevention Service and Collective Public Health, General Directorate of Care to Person, Health and Welfare, Emilia-Romagna Region, Italy; 6 Provincial Laboratory of Microbiology, Local Health Unit of Modena, Modena, Italy

**Keywords:** Cryptosporidium, Drinking Water, small water supply, waterborne infection

## Abstract

*Cryptosporidium* is a leading global cause of waterborne disease, with many reported outbreaks related to main water supplies. In August 2019, an outbreak of cryptosporidiosis involving 80 cases occurred among 114 vacationers in a small municipality located in the Tuscan-Emilian Apennines, north-eastern Italy. After excluding a potential food-borne outbreak, the epidemiological investigation focussed on the hypothesis of a waterborne outbreak. This was confirmed by the finding of *Cryptosporidium* oocysts in stools of the cases and in water samples from the municipal water network. Molecular characterisation revealed the zoonotic species *Cryptosporidium parvum* as the causative agent. A single subtype (IIdA25G1) was found among all cases, and in one of two positive water samples. The municipality’s water supply used spring water that only received a disinfection treatment insufficient to inactivate the parasite. Possible entry means into the water mains were found through further environmental investigations. As these types of water supplies are particularly vulnerable to various environmental factors, a control system based on the risk assessment of each phase of the water supply chain is required to guarantee water safety. Effective methods for detection of protozoan pathogens, which are generally excluded from routine water supply analysis, should be applied.

## Background

The enteric parasite *Cryptosporidium*, along with norovirus, *Giardia*, *Campylobacter* and rotavirus, is among the most frequent causes of waterborne disease [1,2]. In humans, transmission of *Cryptosporidium* occurs via the faecal-oral route, either through direct exposure to infected people (person-to-person infection) or animals (animal-to-person infection), or through ingestion of water (drinking water, recreational water such as swimming pools, water parks, lakes, rivers) or consumption of raw or undercooked food contaminated with infectious oocysts [3]. Infection may remain asymptomatic or manifest as acute gastroenteritis (> 80% of infected individuals). Symptoms occur 1 to 12 days (mean: 7 days) after exposure and usually last 6 to 9 days. The severity and duration of symptoms are linked to the immune status of the host, and cryptosporidiosis can be life threatening in immunosuppressed individuals [4].

There are many *Cryptosporidium* species that can infect humans, but the vast majority of cases are due to *Cryptosporidium parvum*, a zoonotic species that also infects young ruminants, and *Cryptosporidium hominis*, which is essentially only a human pathogen [5]. The environmental route of transmission is of high relevance for *Cryptosporidium* [6]. This is due to several factors including: (i) the high survival rate of oocysts in water (more than 24 months at 20°C), (ii) high resistance to disinfection (30 mg/L of free chlorine are needed to achieve 99% inactivation at pH 7, with a recommended value of 0.2 mg/L for drinking water) [6], (iii) low infectious dose (10–132 oocysts in healthy adults [7]) and (iv) low host specificity [5]. Oocysts lose their infectivity when frozen, boiled or heated over 60°C [6].

The ability of *Cryptosporidium* to survive at high chlorine concentrations [8] and, consequently, at the disinfectant concentrations commonly used in water treatment, has always been a challenge for water treatment plant operators. However, other disinfectants, such as chlorine dioxide, ozone, UV rays and filtration have proved to be rather effective in removing *Cryptosporidium*. Water safety mainly depends on the combination of different treatment stages, and a multi-barrier approach is a key paradigm for ensuring safe drinking water [6]. Nonetheless, in small water supplies managed by local communities that serve only few thousand people, multi-barrier treatment systems are usually not implemented. Thus, in order to ensure the safety of drinking water, more traditional treatments, e.g. disinfection, are used and water quality is checked against certain regulatory parameters.

During 2017–20, 60 waterborne outbreaks of cryptosporidiosis have been detected in Europe, the majority of which involving treated recreational water (swimming pools) as the vehicle of infection [9]. The number of outbreaks linked to contaminated drinking water has shown a notable decrease in the past decades, although, when occurring, large numbers of individuals may be involved, as exemplified by the outbreaks reported in 2010–11 in Sweden [10,11].

## Outbreak detection

On 5 September 2019, a hospital in the province of Modena, north-eastern Italy, notified to the Public Hygiene Service (PHS) of Modena two cases of suspected food-borne disease, which occurred in two children (aged ca 12–14 years). The two children had been hospitalised on 4 September with gastroenteritis. They were part of a group of 99 people (Group A) who had been staying in a municipal hostel in a small town in the Tuscan-Emilian Apennines from 25 to 31 August. In the notification, the hospital informed of the possible presence of further cases of gastroenteritis among other group members.

PHS immediately started the epidemiological investigation and activated the Food Hygiene and Nutrition Service (FHNS). On 6 September, the hostel kitchen and equipment were inspected. The examination of the premises did not support the hypothesis of a food-borne disease. Thus, excluding a food-borne transmission, drinking water appeared to be a potential common source of exposure to pathogens. Faeces from the two hospitalised patients were also tested for *Cryptosporidium* and *Giardia* and the result was positive for *Cryptosporidium* spp. in both cases. The stool test for *Cryptosporidium* spp. was then extended to all Group A members reached by the epidemiologic investigation.

On 11 September, PHS received further notifications of suspected food-borne disease in another group of 15 people (Group B). They vacationed in the same municipality, but stayed in a different lodging facility, from 18 to 25 August. These individuals underwent an epidemiological investigation, which highlighted tap water consumption among the most prominent risk factors. This group was also tested for the presence of *Cryptosporidium* and *Giardia* in faeces. The finding of the same symptoms in different groups of vacationers, whose only common risk factor was tap water consumption, reinforced the hypothesis of a waterborne disease. In August, no other reports of gastroenteritis were received from the area. 

The outbreak was investigated by a task force composed of the Public Health Department and Italian National Institute of Health (ISS), in collaboration with the technicians of the municipality. Here, we describe the investigation of the outbreak, that was supported by the analysis of human and water samples and by environmental data, and consequent results and actions taken.

## Methods

### Setting

The outbreak occurred in a small town in the Tuscan-Emilian Apennines (north-eastern Italy), commonly visited by vacationers in the summer months. The water supply is ensured by networks supplied by springs and managed directly by the municipality.

The epidemiological investigation included the two groups of vacationers who had stayed in the municipality in the periods described above, within which the cases of suspected food-borne diseases had been notified.

### Outbreak case definition

The following criteria were defined: (i) non-case: absence of gastroenteric symptoms (abdominal pain and/or vomiting and/or diarrhoea) and negative diagnostic *Cryptosporidium* stool test, (ii) possible case: presence of gastroenteric symptoms only, with or without fever and (iii) confirmed case: presence of gastroenteric symptoms, with or without fever, and positive diagnostic stool test.

### Epidemiological investigation

The epidemiological investigation was conducted according to the procedures established by the public health department (PHD), which involved two investigation forms.

The first form (used to investigate a food-borne disease case) aimed at gathering detailed information regarding personal data of single cases and contacts, clinical information, food consumption in the 4 days before symptom onset, places of food purchase and consumption, place of residence, potential risk factors and the need for hospitalisation or referral to a doctor. The data were collected through interviews conducted by the PHS health assistants based on a questionnaire, by telephone or in person. No data on comorbidities were collected.

The second form (used to investigate outbreaks related to a common food source) was a summary sheet of the data from the individual interviews and combined information from each single case to allow determination of the attack rate and the epidemic curve.

### Microbiological investigation of human stool samples

All stool samples were tested for rotavirus, adenovirus (immunochromatographic test with kit Rapid Strip Rota-Adeno, Meridian Bioscience Europe) and norovirus (RT-PCR GeneXpert, Cepheid) and for *Cryptosporidium* and *Giardia*. 

The parasitological examination was carried out by direct microscopic observation. For the diagnosis of *Cryptosporidium* spp., an immunochromatographic test (Immuno card stat Crypto/Giardia, Meridian Bioscience Europe) that detects coproantigens of both *Cryptosporidium* and *Giardia* was performed. Positive samples were further analysed by microscopy after staining with the modified Ziehl-Neelsen technique [12].

The first three samples were also examined by a multiplex PCR with the gastrointestinal panel FilmArray GI panel (Biofire), which tests for 22 gastrointestinal pathogens among bacteria (including *Salmonella*), viruses (including rotavirus and norovirus) and parasites.

### Environmental investigation

Environmental investigation included inspections of the suspected water supply system at the springs, tanks, pumps and disinfection plants. Checklists provided and validated by the ISS were used [13]. Results of the analyses of water samples that FHNS draws eight times per year from the water supply system according to the national regulation were re-examined.

As the entry of *Cryptosporidium* oocysts into the water network can be facilitated by rainfall, local rainfall data were gathered by the Regional Agency for Prevention, Environment and Energy for the period of July–September 2019, in order to assess the possible correlation with the epidemic curve.

### Sampling and analyses of water samples

#### Detection of *Cryptosporidium*

The entire procedure of water sampling and analysis was conducted according to the standard ISO 15553:2006 [14]. The sampling was conducted by concentrating 250 L of water on site at the following locations: (i) springs collection tank and pump plant (water influent), (ii) spring collection tank and pump plant (after chlorination), (iii) main tank effluent, (iv) distribution mains sampling point 1 and (v) distribution mains sampling point 2. 

Water concentration was performed using compressed foam filter modules (Filta-Max Xpress Filter Modules, Idexx, United States), followed by elution using the Pressure Elution Station (Idexx) at the ISS. According to the standard, the eluted material was clarified by immune-magnetic separation with anti-*Cryptosporidium* Dynabeads (Dynal, ThermoFisher Scientific), and the samples stained with fluorescent-labelled antibodies (Merifluor *Cryptosporidium*/*Giardia* kit, Meridian Bioscience Europe). The oocysts were enumerated with an epifluorescent microscope (Zeiss), and morphology, size and colour of the particles were assessed with respect to a positive control (Meridian Bioscience Europe).

#### Microbiological analyses

The water analysis for *Cryptosporidium* was carried out in parallel with the analyses of the microbiological parameters established by the regulation of the quality of water intended for human consumption (Directive 98/83/EC as amended and supplemented) [15].

Analyses were performed as follows: Coliforms at 37 °C and *Escherichia coli* according to the ISO 9308–2 [16], enterococci with Enterolert DW, AFNOR validated (Idexx) [17], *Pseudomonas aeruginosa* according to the ISO 16266–2 [18], heterotrophic plate counts (colony counts) at 37 °C and at 22 °C according to the EN ISO 6222 [19] and *Clostridium perfringens* (including spores) according to the ISO 14189 [20].

#### Free chlorine detection

The free chlorine concentration was determined according to the technical manual Rapporti ISTISAN 07/31 [21].

### Molecular characterisation of *Cryptosporidium* in stool and water samples

Stool samples were sent by the provincial laboratory of clinical microbiology of Modena to the ISS, where genomic DNA was extracted from ca 200 mg (or 200 μl in case of liquid stools) using the FastDNA SPIN Kit for Soil and the FastPrep120 apparatus (MP Biomedicals).

DNA from water samples was also extracted from two microscopic slides, which were prepared during the investigation of water samples. Briefly, the surface of the glass slide was scraped with a scalpel and washed with phosphate buffered saline (PBS; 0.02 M, pH 7.4). The collected material was transferred to a sterile 1.5 ml tube and centrifuged at 5,000 rpm for 5 min. After discarding the supernatant, DNA was extracted using the DNA extraction IQ System (Promega).

For the molecular characterisation of *Cryptosporidium*, a first protocol, based on amplification of a fragment of the small subunit rDNA [22], was applied for species identification in human samples. A second protocol, based on amplification of a fragment of the highly polymorphic gene encoding the glycoprotein gp60 protein [23], was applied for subtype identification in both human and water samples. For both protocols, amplification was performed in a final volume of 50 μl containing 25 μl of 2X GoTaqGreen mastermix (Promega), 1 μl of each primer (10 pmol/μl), 5 μl of genomic DNA and 18 μl of nuclease-free water. Negative and positive controls were included in each experiment. Reactions were performed on a PerkinElmer 9700 apparatus (Life Technologies).

PCR conditions for both primary and secondary amplification were as follows: an initial denaturation step of 94°C for 3 min, followed by 35 cycles of denaturation at 94°C for 30 sec, annealing at 50°C for 1 min and extension at 72°C for 1 min, followed by a final extension step at 72°C for 7 min. Positive PCR products were purified using spin columns (QiaQuick PCR purification kit, Qiagen) and sequenced on both strands by a commercial company (BMR Genomics). Sequence chromatograms were edited and assembled using the SeqMan 7.1 software package (DNASTAR). BLAST searches (http://blast.ncbi.nlm.nih.gov/Blast.cgi) against the GenBank database were used to identify *Cryptosporidium* at the species and subtype levels.

## Results

### Descriptive epidemiology

One hundred and fourteen people were involved in the outbreak, namely 99 individuals belonging to Group A (57 males and 42 females, median age: 12 years, interquartile range (IQR): 7) and 15 members of Group B (10 males and 5 females, median age: 39 years, IQR: 39). One hundred and two individuals were available for an interview (Group A, n = 88; Group B, n = 14), and this revealed that 80 (Group A, n = 69; Group B, n = 11, corresponding to an attack rate of 78% for both groups) had gastroenteric symptoms between 18 August and 5 September. Two people were hospitalised and two people were treated in the emergency room. The incubation period (calculated as the time between arrival to the locality and the onset of symptoms, since all members of the two groups consumed tap water) ranged from 8 h to 13 days (median: 6 days), while the duration of symptoms ranged from 20 h to 16 days (median: 3 days). The case with an 8-h incubation tested positive also for other pathogens (*Salmonella* and rotavirus).

A stool test for the detection of *Cryptosporidium* and *Giardia*, rotavirus, adenovirus and norovirus was made available to all the interviewees at the provincial laboratory of clinical microbiology of Modena. Stools samples were collected from both groups between 5 and 12 September. Of the 87 stool samples tested (Group A, n = 76; Group B, n = 11), 75 (corresponding to 86%) were positive for *Cryptosporidium* (Group A, n = 67; Group B, n = 8). Among these, 5 tested positive also for *Giardia* (Group A, n = 4; Group B, n = 1), 1 for rotavirus (Group A), 1 for rotavirus and *Salmonella* (Group B), and 2 for norovirus (Group A, n = 1; Group B, n = 1).

**Figure 1:**
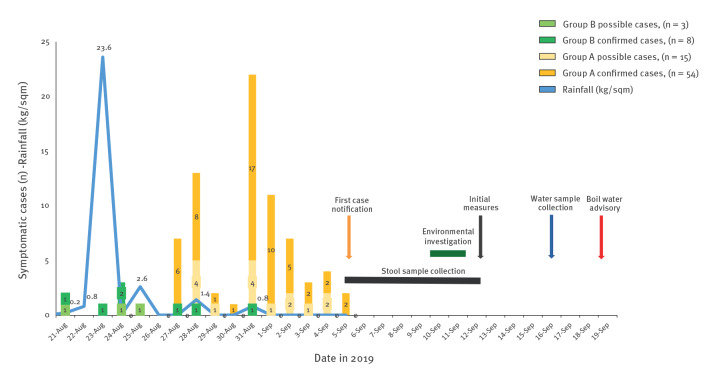
Epidemic curve of cryptosporidiosis cases and local rainfall data, municipality of Tuscan-Emilian Apennines, Italy, August–September 2019 (n = 80)

The epidemic curve and the rainfall data are reported in Figure 1.

Symptomatic cases from Group A and Group B, which include both possible (n = 18) and confirmed cases (n = 62, positive stool test), are represented. Cases are shown on the day of onset of symptoms. One confirmed case in Group B showed onset of symptoms on 18 August (the same day of the arrival; case with 8 h incubation) and is not visible in the figure but included in the total n.

Local rainfall data were gathered by the Regional Agency for Prevention, Environment and Energy.

### Environmental investigation

**Figure 2:**
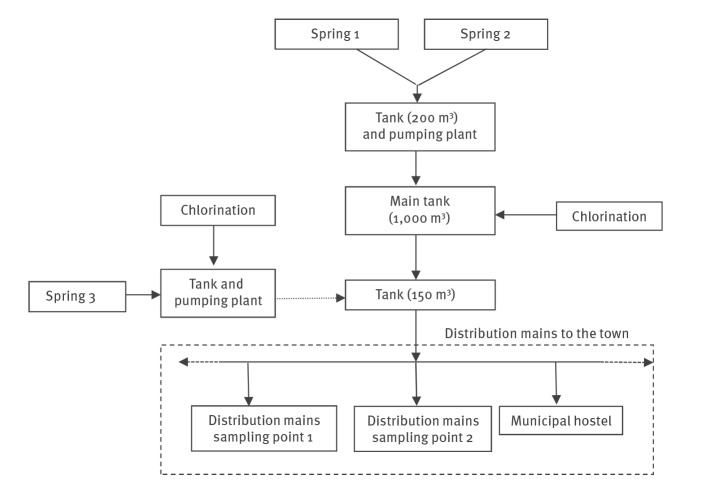
Flowchart depicting the water distribution system, municipality of Tuscan-Emilian Apennines, Italy, September 2019

Environmental inspections were conducted on the local water supply system on 10 and 11 September. The water main, managed by the municipality, supplies ca 1,000 inhabitants and draws from two main springs (Figure 2). The first spring (Spring 1) is located in a wooded area, ca 710 m above sea level, with no human settlements nearby. The catchment was in good structural condition and adequately protected from infiltration from the soil surface. The second spring (Spring 2) is also located in an uninhabited wooded area, ca 650 m above sea level. It was found in poor condition, without a protection area, with stagnant water in the catchment and risk of run-off.

Continuous chlorination with sodium hypochlorite (target value: 0.2 mg/l).

Spring 3 was connected to the water distribution plant until April 2019.

The water from the two springs flows into a 200 m^3^ tank, which was in fairly good condition. The water is then pumped into the main tank of 1,000 m^3^, at 920 m above sea level, where the water is treated with sodium hypochlorite (continuous chlorination, target value: 0.2 mg/l). Before supplying the local distribution mains, the water flows from the main tank into a second tank of 150 m^3^. This tank was old and buried in the ground, only accessible from a hatch and not subjected to periodic cleaning procedures. Until April 2019, this tank was also supplied by a third source (Spring 3) located on an agricultural land and exposed to possible contamination from a nearby livestock farm. This spring was disconnected for technical reasons.

In June 2019, analytical controls according to the Italian legislation [24] showed the presence of *Clostridium perfringens* (25 CFU/100 ml) in distribution mains. This finding was notified to the municipality, as usual for all non-compliant results.

### Water sample analysis

On 16 September, three water samples were collected at the effluent of the main tank and at two points of the distribution systems. The results of the bacteriological/parasitological analyses are reported in Table 1.

**Table 1:** Results of the analysis of collected water samples, municipality of Tuscan-Emilian Apennines, Italy, 16 September 2019 (n = 3)

Sample location	Coliform bacteria at 37°C^a^	*E. coli*	Enterococci	*P. aeruginosa* ^b^	*C. perfringens* (including spores)	Colony count (37°C and 22°C^c^)		*Cryptosporidium* spp.^d^	Free chlorine^e^
	MPN/100 ml				CFU/100 ml	CFU/1 ml		n of oocysts /100 L	mg/L
Main tank effluent	4	< 1	1	< 1	16	4	14	NA	0.21
Distribution mains sampling point 1	< 1	< 1	< 1	< 1	< 1	6	5	35	0.20
Distribution mains sampling point 2	< 1	< 1	< 1	< 1	< 1	2	221	269	0.40

C: *Clostridium*; CFU: colony-forming unit; E: *Escherichia*; MPN: most probable number; NA: no analysis performed; P: *Pseudomonas*.

^a^ Coliform bacteria at 37°C, *E. coli*, enterococci, *C. perfringens* (including spores): 0/100 ml.

^b^ *P. aeruginosa*: No value defined by regulation.

^c^ Colony count at 22°C: No abnormal change in 1 ml.

^d^ *Cryptosporidium* spp.: 0/100 L.

^e^ Free chlorine concentration (recommended value): 0.2 mg/L, Safety cut-off for drinking water [24].

### Molecular characterisation of *Cryptosporidium* in stool and water samples

Genomic DNA was extracted from 71 of the 75 stool samples, from both Group A and B, sent from Modena Microbiology Laboratory to the ISS, whereas the remaining four samples were discarded because of an insufficient amount of material. PCR and sequencing of the small subunit rDNA revealed the presence of *Cryptosporidium parvum* in eight randomly chosen stool samples. To determine the *C. parvum* subtype(s) associated with the outbreak, all genomic DNA samples (n = 71) were subjected to PCR for the amplification of the gp60 gene, and 56 (79%) tested positive. Sequencing of 16 randomly chosen PCR products showed the presence of a single subtype, IIdA25G1, with 100% homology to a human-derived sequence from France (GenBank accession number, KT716860).

The two genomic DNA samples extracted from the two microscopic slides which were prepared during the investigation of water samples also tested positive by PCR at the gp60 locus. Sequencing revealed the presence of the IIdA25G1 subtype from the slide representing the distribution mains sampling point 1 and of another subtype, IIaA15G2R1, from the slide representing the distribution mains sampling point 2.

## Outbreak control measures

On 12 September 2019, preliminary results of the epidemiological survey were presented to the municipal administration and initial measures were called for, including provisions of bottled water for children and hospitalised patients over 65 years of age, pending the results of the analyses of water samples. On 19 September, after the identification of *Cryptosporidium* oocysts in the distribution water supply, the local health authority issued a boil water advisory.

Immediately thereafter, the task force set up by the municipality implemented the following measures: (i) analytical controls on the microbiological quality of the spring water, (ii) suspension of supply from springs with non-compliant analytical data and in a poor state of maintenance, (iii) extraordinary cleaning and sanitisation of the tanks, (iv) additional chlorination at the tank collecting water from Springs 1 and 2 and (v) sanitisation of the pipelines from the collection tank to the main tank, and of the distribution pipes.

Water samples for microbiological analyses were collected again on 20 November 2019. The results are reported in Table 2.

**Table 2:** Results of the analysis of collected water samples, municipality of Tuscan-Emilian Apennines, Italy, 20 November 2019 (n = 5)

Sample location	Coliform bacteria at 37°C^a^	*E. coli*	Enterococci	*P. aeruginosa* ^b^	*C. perfringens* (including spores)	Colony count (37°C and 22°C^c^)		*Cryptosporidium* spp.^d^	Free chlorine^e^
	MPN/100ml				CFU/100ml	CFU/1ml		n of oocysts /100 L	mg/L
Spring collection tank and pump plant (water influent)	6	3	< 1	< 1	0	35	93	NA	Not detectable
Spring collection tank and pump plant (after chlorination)	201	95	1	9	0	40	471	0	0.10
Main tank effluent	< 1	< 1	1	< 1	0	1	9	0	0.47
Distribution mains sampling point 1	< 1	< 1	< 1	< 1	0	1	0	0	1.44
Distribution mains sampling point 2	< 1	< 1	< 1	< 1	0	0	32	0	0.26

C: *Clostridium*; CFU: colony-forming unit; E: *Escherichia*; MPN: most probable number; NA: no analysis performed; P: *Pseudomonas*.

^a^ Coliform bacteria at 37°C, *E. coli*, enterococci, *C. perfringens* (including spores): 0/100 ml.

^b^ *P. aeruginosa*: No value defined by regulation.

^c^ Colony count at 22°C: No abnormal change in 1 ml.

^d^ *Cryptosporidium* spp.: 0/100 L.

^e^ Free chlorine concentration (recommended value): 0.2 mg/L, Safety cut-off for drinking water [24].

On 19 December, after confirmation of acceptable water quality, the boil water advisory was lifted and implementation of a water safety plan was initiated in accordance with the recommendations by the World Health Organization (WHO) [25].

## Discussion

Here, we describe a human outbreak of cryptosporidiosis in Italy with a high attack rate, which occurred in a municipality of the Emilia-Romagna region, north-eastern Italy. We were able to link the outbreak caused by *C. parvum* to a small local water supply from springs managed directly by the municipality. 

Cryptosporidiosis is a notifiable disease under surveillance at the European level. In the latest report from the European Centre for Disease Prevention and Control (ECDC), 14,252 cases were officially reported by 26 European countries through the European Surveillance System (TESSy), with higher prevalence rates in Northern European countries and among children aged 0–4 years [26]. However, Italy, as well as Austria, Denmark and France, do not yet report data about cryptosporidiosis, as cases are not subject to mandatory notification. Consequently, this parasitic infection remains under-diagnosed and under-reported in Italy, with limited epidemiological information mostly derived from historical surveys in immunocompromised individuals or some case reports. 

In Italy, interestingly *C. parvum* – and not *C. hominis* – has been associated with most human cases [27]. Indeed, more data are available on the prevalence and species/genotypes of *Cryptosporidium* circulating in animals, particularly livestock and pets [28], including many reports on *C. parvum*. Studies on the occurrence of *Cryptosporidium* spp. in water are also limited; the few environmental surveys have demonstrated that the parasite is present in wastewater and surface waters, but it has not yet been detected in drinking water [29-33]. Little is known about the genetic identity of the parasites, although both *C. parvum* [33] and a non-zoonotic genotype from wild rodents [31] were identified in these surveys. Before the outbreak reported here, a single waterborne outbreak of *Cryptosporidium* has been reported in Italy, involving 294 individuals of a drug rehabilitation community of 1,731 members in the Emilia-Romagna region in 1995. Environmental investigation detected oocysts only in sediments collected in water reservoirs, whereas they were not found in the water itself [34].

As mentioned, a trend towards a decrease in the number of outbreaks linked to drinking water has been observed in Europe in the last two decades [9], likely because of improvements in water quality through the closure of or installation of filtration systems at previously unfiltered water supplies [35]. Nonetheless, small community water supplies can often represent a challenge for the local health authorities. Paradoxically, a compliant microbiological result following a routine test procedure might induce an underestimation of the threat posed by more resistant pathogens, such as *Cryptosporidium.* Useful information for risk assessment may be indirectly derived from the detection of *Clostridium perfringens*, which shares similar characteristics with some protozoa, such as the high survival rate and the ability to generate spores that are resistant to oxidant agents.

In this context, the presence of *Clostridium perfringens* in the water samples collected in the distribution system in June 2019 and in the tank effluent on 16 September 2019 is of particular significance for the specific characteristics of the bacterium [36]. The temporal correlation between the intense rainfall (23 kg/sqm) on 23 August 2019 and the peak of the epidemic curve, which spanned the period from 28 August to 1 September 2019, is also particularly striking. Moreover, the inspections allowed the detection of poor spring protection from possible environmental contamination (including that from animal origin), together with the condition of the catchments, which in turn might cause run-off.

The possibility to analytically detect *Cryptosporidium* in water after having verified its presence in the faeces of infected individuals allowed the formulation of a clear hypothesis on the causes of the outbreak. The detected concentrations of the pathogen were low (0.35–2.69 *Cryptosporidium* oocysts/L), in comparison to the usual 2 L/day water consumption per inhabitant; however, considering the low infectious dose of *Cryptosporidium* [7], these values may still be considered relevant. In other waterborne outbreaks of cryptosporidiosis, even lower levels of oocysts (less than 1 oocyst/10 L) have been detected [10]. Other factors, including oocyst viability, the genetic identity of the parasite, and immunity of those exposed, should be considered in addition to the contamination level, as these factors affect the likelihood of an outbreak occurring [37]. The time elapsed between the onset of cases and water sampling (ca 1 month) supports the hypothesis that oocysts were present both in the tanks and in the distribution mains, possibly inside the biofilm. Heavy rain and other adverse weather phenomena can also lead to a further worsening of the quality of water sources by enhancing the spread of pathogens.

The molecular characterisation of stool samples revealed that the zoonotic species *Cryptosporidium parvum* was responsible for the outbreak. Subtyping at the commonly used gp60 gene marker showed the presence of subtype IIdA25G1. This subtype has been previously identified in human samples from France, the United Kingdom and New Zealand, as well as in goat kids from Spain [38] and in calves from Romania [39]. The same subtype was identified in one of the two water samples (Distribution mains sampling point 1), supporting the waterborne origin of the infection. Interestingly, the more common *C. parvum* subtype, IIaA15G2R1, was identified in the other water sample (Distribution mains sampling point 2). The correlation between the clinical features and the presence of the waterborne parasite is then particularly strong.

The measures for the decontamination and sanitation of the pipelines and the tanks taken by the municipality allowed full recovery of the water quality. However, further investigation is needed to detect the origin of the contamination. Considering the characteristics of *Cryptosporidium* illustrated above, we can assume that the contamination of the water supply system dates back earlier than April 2019. Contamination of the pipes with oocysts may have been linked to the use of Spring 3, given the possibility of manure contamination from nearby agricultural activities. The poor condition of Spring 2 may also have played an important role.

Of note, cases were found in vacationers only, and not in the residents of the municipality. There is evidence that *Cryptosporidium* infection induces a protective immune response [4,40,41], and that previously exposed individuals mostly develop asymptomatic or light infections, which are often undiagnosed, while naive individuals are more susceptible to severe forms of the disease.

The WHO Guidelines, which lay down the Water Safety Plan approach [42], stress that the quality of the supplied water can only be ensured through a systematic approach that includes risk assessment throughout the entire water supply chain, together with the availability of analytical methods to test the effectiveness of the implemented control measures. The new European Directive (EU) 2020/2184 [43] introduced the principles of the Water Safety Plan, which will have to be transposed into national legislations by each Member State in order to provide safe water and guarantee consumers’ health.

This study has some limitations. Firstly, the time passed between the onset of symptoms and the laboratory diagnosis of the infection may have caused an underestimation of the number of infected individuals. Secondly, the epidemiological survey was not designed to identify statistically significant correlations between exposure to the main risk factor (consumption of tap water) and symptoms, but rather to identify symptomatic cases and request laboratory tests. Finally, it was not possible to distinguish primary cases from possible secondary cases and no information was collected on coexisting pathological conditions that could have facilitated the onset of symptoms.

## Conclusions

This investigation underscores that, in the case of small water supply systems, risk assessment and implementation of protective barriers represent an additional, fundamental condition for the provision of safe drinking water. An improvement in the recording and reporting of cases of cryptosporidiosis in Italy will contribute to further progress in surveillance, including timely recognition and management of outbreaks.
